# Diet in Protracted Fevers

**Published:** 1852-02

**Authors:** E. B. Haskins


*Diet in Protracted Fevers.* By E. B. Haskins, M. D.—It is well known to our readers, who have at all kept pace with the advancement of chemico-physiology, that, in the food of man, there are two great classes of alimentary substances, to subserve the wants of the ever changing organism—the *nitrogeneous* and *non-nitrogeneous.* The former being destined for the nourishment of the tissues, whilst the latter subserves, mostly, the purpose, by slow combustion, of the generation of animal heat. It is not denied that, in the process of disintegration, or wasting of the tissues, some heat is evolved, also, that some fat enters into the composition of those tissues, whilst other portions seem necessary to give symmetry to the body and form cushions of support to moveable parts; yet the above classification of alimentary substances, based upon their uses, is regarded mainly as true. It may also be remarked, that that portion of the non-azotized substances, not directly burned off in the circulation, is converted into fat, and deposited in the adipose tissues for future use.

This economical disposition of respirable materials, is beautifully illustrated in the habits of hybernating mammalia—that go into their winter retreats loaded with fat, and come out in the spring comparatively lean. As such animals remain physically inactive during the season of hybernation, of course but little waste of muscular or other vital structures takes place—not more than can readily be supplied by the proteinaceous compounds already in the vascular system.

Now, when a subject enjoying ordinary health is seized with continued fever, but little or no food is required for a number of days; the blood being charged with azotized matter sufficient to supply the waste from tissual disintegration, whilst, in the adipose cells, there is already fat enough for respiratory purposes.

But should the disease continue unchecked, the time arises when food becomes imperative—when the limbs become lean and emaciated, and prostration of strength supervenes. All agree as to the *time* for the more prompt and regular administration of food; but the *kind* of food best suited for this stage seems not to have met with so general an agreement. The diet usually prescribed in such cases is animalized waters, as beef tea, chicken water, &c., and the manner of preparing them is to remove all of the fat as it rises to the surface in the process of boiling. So the patient, it is perceived, ingests nothing but azotized food.

Now what, *à priori*, will be the course of a protracted fever under such a dietetic system? It is clear—the adipose tissue being exhausted of its moveable fat, and no starch, gum, sugar, or other combustible material being furnished to the blood, the oxygen of the inspired air seizes upon the tissues, and the brain, being, perhaps, the most oxidizable, is the most energetically attacked; and as fatty matter enters largely into its composition, no adequate reparation can take place; hence delirium, subsultus tendinum, wakefulness, and other morbid cerebro-spinal activity—phenomena too often witnessed at the bedside, and always portend an unfavorable issue.

This hypothetical view of the pathology of the brain is, in some degree, supported by the fact that post-mortem examinations have failed to detect any constant lesion in that organ; and when congestion or inflammation has been found, it is not violent to presume that it came on as a secondary lesion.

The point in the dietary of protracted fevers, to which I wish to direct attention, is already clear to your mind—that non-nitrogenized substances, as starch, gum, sugar, &c., should be freely administered, instead of the exclusively nitrogenized diet. By thus furnishing respirable materials, the tissues, particularly the *brain*, are protected from the destructive influences of the inspired oxygen. These materials, then, are far more essential to the organism, under such circumstances, than the azotized substances, since, whilst the muscular system is comparatively at rest, the histological elements undergo very slow disintegration, as is exemplified in hybernating animals.

The non-azotized alimentary substances, as is well known, can be rendered as palatable and inoffensive to the sick (oils excepted) as can the nitrogenized. Sweetened gum water, arrowroot-jelly, barley water, and even the gruel of Indian meal are quite palatable and unirritating to the most delicate stomach. To secure, however, all of the possible dietetic wants of the system, they may be alternated with the nitrogenized diet.

In conclusion, I will take occasion to remark, that, in determining a course of practice upon theoretical grounds, great caution is necessary, that we commit no extravagances. We should never, from *a priori* reasoning alone, abandon well-established therapeutic usage. The most that may be ventured upon with impunity, is the modification and correction of unenlightened experience, and the furnishing materials for such blanks as observation has failed to fill up. Ratiocination is too uncertain a guide to be wholly trusted to in the prosecution of an art like therapeutics, based upon *progressive* science. Kept within these restricted bounds, reason performs her legitimate office in the advancement of a profession that can never be purely empirical or rational.—*Nashville Journal of Med. and Surg.*, Dec. 1851.

